# Anticipation training for expert tennis players when facing a specific player

**DOI:** 10.3389/fpsyg.2024.1508627

**Published:** 2025-01-07

**Authors:** Céline Triolet, Nicolas Benguigui

**Affiliations:** ^1^Université Paris-Saclay CIAMS, Orsay, France; ^2^Université d'Orléans, CIAMS, Orléans, France; ^3^Université de Caen Normandie, EA4260 CESAMS, Caen, France

**Keywords:** anticipation training, contextual information, tennis, implicit protocol, experts

## Abstract

**Introduction:**

In fast ball sports, such as tennis, when spatiotemporal constraints are high, players have to anticipate the opponent action. Not much is known about how players acquire and improve this ability. The aim of this study was to use an implicit training protocol (no information was given to participants) based on the knowledge of one particular opponent to analyse how experts could improve their anticipation ability.

**Method:**

Professional tennis players were tested and trained in a protocol consisted of watching videos with temporal occlusions before the opponent stroke and guessing the direction of the stroke. Three groups took part in the experiment: one with a specific training with the same opponent as in pre- and post-tests; one with a various training with players other than the one used in pre- and post-tests (to control that the improvement is link with the knowledge of one specific opponent and not to an adaptation to the task) and one control group without practice.

**Results:**

Only the group trained with the same opponent increased its response accuracy.

**Discussion:**

Our results suggest that anticipation can be improved in professional tennis players with a very specific training providing information about the opponent.

## 1 Introduction

In ball sports, experts have to produce their actions under a tight time pressure. In some situations, they don't have enough time to wait for a complete information to decide what response to provide (Triolet et al., 2013). In such situations they produce anticipations based on partial information and their knowledge of the game and of their opponent. Therefore, perceptual-cognitive skills are a key attribute of expertise in fast ball sports such as tennis (Williams et al., 2011). One of the questions that arises in this field is how these anticipation skills are learned and can be improved through training. In this article we aim to show that it is possible to improve this ability for expert tennis players against a specific opponent using a short training protocol based on video footages.

It appears that three main factors determine anticipation skills. First, experts are better than novices to recognize sport-specific patterns (North et al., 2011). Secondly, they can better use kinematic information from their opponent when he/she is preparing is action (Farrow et al., 2005). Thirdly, regarding the use of contextual information, the findings from previous studies are more mixed. Indeed, depending on the type of contextual information used [such as knowledge of the opponent (McRobert et al., 2011), the score (Farrow and Reid, 2012), or the relative positioning of players during the point (Loffing and Hagemann, 2014)], it appears that experts can access to this information, but sometimes intermediate players or even novices can as well. For example, Farrow and Reid (2012) demonstrated that only experts were able of using the score to enhance the relevance of their anticipations, whereas the positioning of opponents and teammates was a source of information accessible to intermediate-level players (Paull and Glencross, 1997). The use of knowledge about the opponent and their playing habits seems to be limited to expert players (McRobert et al., 2011). Finally, the use of the relative positioning of players during the point appears to depend on the quality of the contextual information available (Triolet et al., 2022). If anticipation is admitted as a key determinant of expertise, the training of this skill appears to be an essential question for performance in fast ball sports (see Zentgraf et al., 2017 for a review). Protocols were mostly based on occlusion paradigms (Farrow and Abernethy, 1998) and two different performance factors are generally used to analyze protocol-related improvement, with the decrease of reaction time and/or the improvement of response accuracy.

Concerning response accuracy, Scott et al. (1998) showed an improvement after a training protocol for intermediate tennis players to return a serve. The task was to predict the bounce location of the serve that was occluded at the impact. During training sessions, participants were shown videos of the serve in slow motion (see for more examples, Abernethy et al., 1999; Alsharji and Wade, 2016).

Concerning the decrease of response time, Farrow and Abernethy (1998) conducted a study in which tennis beginners were taught to pay attention to certain elements considered important for anticipation such as the position of the server's feet, the ball's delivery, the position of the racket and its speed. After eight 15-min training sessions in which the players saw videos of various serves from “good players” with temporal occlusions, the trained group significantly improved their response times (see Williams et al., 2003 for more examples).

Finally, Moreno et al. (2002) trained recreational tennis players and analyzed the evolution of accuracy and reaction time. Results mainly showed exchanges between response accuracy and response time. For some participants, a decrease in response time was associated with a decrease in response accuracy while for other participants, the inverse evolution was observed. There are therefore inter-individual differences in learning and the priority between time and accuracy of responses may vary.

Researchers have also investigated the impact of various instructional methods and techniques to direct participants' attention. For instance, Smeeton et al. (2005) conducted an experiment in tennis involving four distinct groups: an explicit group that received prescriptive information, such as the location of relevant advance cues and their impact on performance; a guided discovery group that was informed about the location of key postural cues and encouraged to deduce the relationship between body position and shot outcome; a discovery group that was prompted to explore the relationship between perceptual cues and shot outcomes independently; and a control group that received no instruction. Participants undertook two different tasks: a laboratory task and a field task. All three intervention groups showed performance improvements from pre-test to post-test. However, the explicit group's improvements appeared less robust under pressure. Additionally, the explicit and guided discovery groups demonstrated faster performance improvements during training compared to the discovery group.

The research indicates a predominant focus in training protocols on enhancing the pick-up of kinematic cues (Smeeton et al., 2005; Abernethy et al., 2012), with comparatively fewer studies concentrating on pattern recall (for an exception see Schorer et al., 2018) or the use of contextual information (for an exception see Gray, 2015). Broadbent et al. (2015) recommended integrating contextual information into training protocols to better reflect real-world performance demands. Triolet et al. (2013), in an *in-situ* analysis of expert tennis players, further demonstrated that the use of contextual cues significantly improved anticipation accuracy.

Furthermore, Loffing and Cañal-Bruland (2017) raised an important question concerning the optimal methods for communicating situational probabilities to athletes, pointing toward the need for more effective instructional strategies in training. Given that anticipation often involves implicit processes, it is plausible that implicit learning protocols might provide distinct advantages. Farrow and Abernethy (2002) explored this possibility by comparing explicit and implicit methods. A progressive temporal occlusion paradigm was employed to assess players' abilities to predict the direction of an opponent's serve on the tennis court before and after training. Players were instructed to respond either by attempting a return of serve or verbally predicting the serve's direction. The study included four groups: (a) an explicit learning group, which watched videos of serves accompanied by a tennis instructor explaining the relationships between key biomechanical cues and serve direction; (b) an implicit learning group, which watched the same videos without receiving any explicit information but were instead tasked with estimating the ball's speed during the serve; (c) a placebo group, which received no instructional input beyond watching tennis videos; (d) a control group, which did not watch any videos. The findings showed that only the implicit learning group demonstrated a significant improvement in the accuracy of their serve direction predictions following the training.

Wulf and Weigelt (1997), in a study aimed at developing a complex motor skill on a ski simulator, provided another definition of learning protocols. They distinguished explicit learning, where participants are given explicit information or instructions to aid task performance, and implicit learning, where participants are informed only of the task's goal, without any guidance on how to achieve it.

Finally, it is interesting to underline that only few training research studies have been conducted with experts (for exceptions, see Fadde, 2016 who ran a training protocol during the entire season with a college baseball team or Alsharji and Wade (2016) who ran a training protocol with elite and national youth handball goalkeepers).

In this context, the aim of this study was to use an implicit training protocol according to the definition from Wulf and Weigelt (1997) to determine whether a specific anticipation training protocol facing one specific opponent enable experts to improve their capacity to predict his actions. Three groups took part to the protocol. The first group followed an anticipation training in order to improve their ability to predict the shots of a specific player (same opponent Group). The second had the same training protocol with different players to check if the expected improvement of the first group was not due to an adaptation to the task (various opponents Group). The third one was a control Group without training session.

We hypothesized that the same opponent Group should improve both their percentage of correct responses and their response speed, as the result of taking better account of their opponents' playing preferences (McRobert et al., 2011). Considering that we worked with expert players and that tactical information to enhance anticipation skills can be used even by non-expert players, it is plausible that this information has already been acquired by our expert players and, therefore, will not contribute to further improvements in their anticipation (Triolet et al., 2022). This is why no improvement was expected for the various opponents Group.

## 2 Methods

### 2.1 Participants

Thirty-nine male expert players (mean age: 28.1 ± 9.92 years old) took part in this experiment. They were all international or national players and practiced tennis since 17.38 ± 8.5 years on average. They were ranked between 4.5 in the American ranking and the top ten ATP ranking. They have been randomly divided into three groups of 13 players: the same opponent Group, the various opponents Group and the control Group. The three groups were equivalent in terms of tennis experience and age (Table 1). Participants took part voluntarily in the experiment and written and informed consent were obtained. The research received ethical approval from the lead institution.

**Table 1:** Participants' characteristics according to the different groups.

	**Mean age (years)**	**Average number of years of tennis practice (years)**
Same opponent group	28.15 (±10.82)	18.62 (±9.36)
Various opponent group	26.62 (±10.44)	15.38 (±7.34)
Control group	29.38 (±9.03)	18.15 (±8.99)

### 2.2 Materials

#### 2.2.1 Clips and task

One hundred and sixty-eight video clips were obtained from broadcast male ATP tennis games, filmed in the longitudinal axis of the court. Each clip ended with a winning shot (could be any tennis shots, except serves) delivered by the player filmed at the top of the screen from a frontal perspective. The winning shot occurred on the 4th stroke of the rally in a very unfavorable situation for the opponent. This kind of shot was selected to increase the need to anticipate for the participant (Triolet et al., 2013). The occlusion moment was set 340 ms before ball/racket contact in order to avoid providing participants kinematic information related to shot outcome (Farrow and Abernethy, 2003). Participants had to indicate where the occluded shot would be played, either on the right or left side of the pitch, by pressing a corresponding button on a keyboard.

An expert reference player has been chosen to make the pre-test and post-test clips for all groups. He has been ranked ATP No. 4 and was professional until 1999. The pre-test and post-test consisted of 12 trials in which the expert reference player made a winning shot. No feedback was given to the participants after the clip.

After the pre-test, the same opponent Group had a training phase composed of 72 trials in which the expert reference player made a winning shot. Then they finished with the post-test. The various opponent Group was also doing the pre- and post-test facing the expert reference player. However, during the training phase, they were confronted to twelve different players other than the expert reference player. These players have been ranked between ATP Nos. 1 and 12. The clips were selected on the same principle with a winning shot occurring after four shots. This group had the same amount of trials. The control Group only realized the pre-test and post-test without feedback.

#### 2.2.2 Procedure

Video clips were presented on a 17″ laptop (Dell, Round Rock, TX, USA) using a specific software (E-Prime, Psychology Software Tools, Inc., Pittsburgh, USA). Participants sat 40 cm from the laptop's screen. The experiment was run in one session and the total duration of the experiment was ~1 h for training groups and 15 min for the control group. The response accuracy (right/left) and the response time for each clip were recorded by the E-prime program.

**Figure 1:**
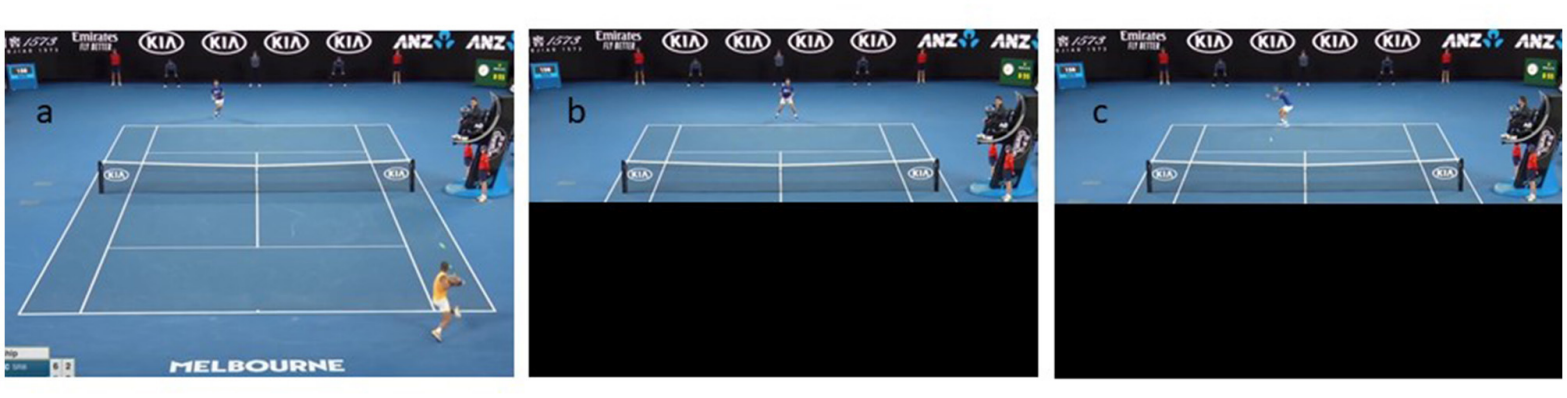
Illustration of the procedure of one trial. **(A)** Shows the frozen image at the beginning of the clip. The clip unfolds. **(B)** Shows the rectangle that hides the bottom player after his last shot. **(C)** Shows the last image before total occlusion and the participant's response.

Each trial began with a countdown from 3 to 1 before starting with a freeze frame of the first image. Then the video began 200 ms before the first stroke of the sequence (Figure 1A) and ended 340 ms before the 4th stroke hit by the player at the top of the screen (Figure 1C). To avoid participants being influenced by the behavior of the player at the bottom of the screen (i.e., player to whom the participant had to substitute himself), the player was hidden by a black rectangle 200 ms after his last stroke (Figure 1B).

When occlusion occurred, the screen turned black and the participant had 1 s to indicate if the opponent played a winning shot directed on the right by pressing the key “P” or on the left side of the court by pressing the key “A”. For the pre-test and the post-test series, a new trial began immediately after the response. For the training series for training groups, feedbacks were given to the participant.

The experiment began with a familiarization session containing 12 trials with various opponents. After this, the participant had to respond to the pre-test series containing 12 trials with the same player. The same opponent and various opponents groups were then facing six training series of 12 trials each. Finally, the post-test series containing 12 trials was conducted. Training series and clips inside each series were presented in a randomized order.

During the training session, two kinds of feedback were given: first, the participant was told whether his response was right or wrong; second, he watched the sequence again without occlusion to allow him to pick up additional information. No feedback was provided by the experimenter. As soon as the clip with the response was finished, a new trial was started. At the end of each block, feedback relating to the percentage of correct responses for each block was provided to keep the participant's interest and to encourage him to improve it.

### 2.3 Data analysis

For each participant, the percentage of correct responses and the mean response time were calculated as dependant variables.

No data were excluded by the experimenters. However, there were 52 trials out of 936 in which participants took too long to respond (more than 2 s) and for which no values were recorded.

Data were analyzed using two-way factorial repeated measures ANOVAS with Groups (the same opponent Group, the various opponents Group and the control Group) as a between-participants factor and Tests (Pre and post tests) as within-participants factors. The obtained percentages of correct responses were transformed to Fisher *z*-scores (Fisher, 1942) in order to run ANOVAS. The significance level set for the statistical analysis was *p* < 0.05.

## 3 Results

### 3.1 Percentage of correct responses

**Figure 2:**
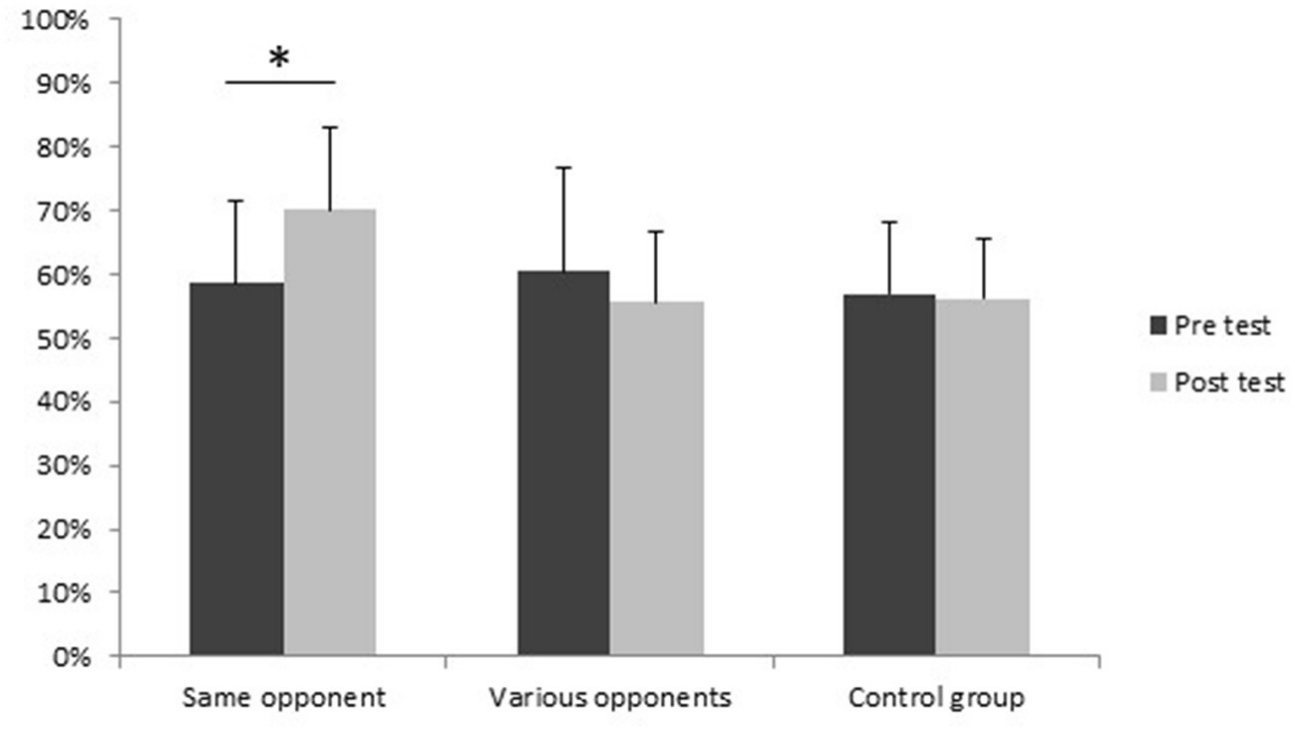
Mean percentage of correct responses for pre and post-tests for each group. *Improved response accuracy between pre- and post-tests for the same opponent Group (*p* < 0.05).

*ANOVA* on mean percentage of correct responses revealed a main effect for Groups, [*F*_(2, 36)_ = 4.005, *p* < 0.05, η^2^ = 0.182] and an interaction effect between Groups and Tests [*F*_(2, 36)_ = 3.528, *p* < 0.05, η^2^ = 0.164] (Figure 2). Newman-Keuls *post-hoc* tests on the interaction showed that percentages of accuracy were not different for the pre-test between the three groups. The percentage of correct responses of the same opponent Group significantly increased with training, while it was not the case for the others groups. For the post-test, the percentage of correct responses of the same opponent Group is significantly different from the percentages of correct responses obtained by this group in the pre-test and by the control and the various opponents Groups in both the pre-test and the post-test.

### 3.2 Response time

*ANOVA* on response time revealed no effect for Groups, [*F*_(2, 36)_ = 0.976, *p* > 0.05] and no interaction effect between Groups and Tests [*F*_(2, 36)_ = 1.730, *p* > 0.05]. However, *ANOVA* revealed a main effect for Tests [*F*_(1, 36)_ = 20.087, *p* < 0.05, η^2^ = 0.358]. The mean response time for all participants decreased from 669 ms (±77) to 495 ms (±84) for the pre- and post-test, respectively (Table 2).

**Table 2:** Mean response time for each group.

	**Pre test response time (ms)**	**Post test response time (ms)**
Same opponent group	734.82 (±296)	463.59 (±257)^*^
Various opponents group	584.42 (±229)	431.24 (±221)^*^
Control group	687.34 (±327)	589.36 (±282)^*^

^*^Shows a significant difference between the mean response time in pre-test and post-test.

## 4 Discussion

The aim of this study was to evaluate if an implicit perceptual training protocol could enable expert tennis players to better anticipate the action of a specific opponent. Results support this hypothesis with an increase of response accuracy in the same opponent Group. The lack of improvement for the other groups suggests that this improvement is due to this specific training and not to a familiarity effect with the protocol (Williams and Grant, 1999). This result is consistent with previous studies which showed that the knowledge of the opponent can improve expert players anticipation: the opponent preferences (Gredin et al., 2020), the opponent laterality (Loffing et al., 2016) or the knowledge of the opponent level of play (Huesmann and Loffing, 2019).

Concerning the various opponents Group, it seems they already have the capacity to use the relative position of players to anticipate and this training protocol didn't help them to improve their anticipation judgments.

Regarding the response time, a decrease was recorded for all groups. So we can conclude that there is a familiarity effect to the protocol. The advantage of training with the same opponent did not appear on response time but only on response accuracy (Scott et al., 1998; Abernethy et al., 1999).

The origin of the improvement of response accuracy in the same opponent Group can be questioned. Our protocol used an implicit method leaving open speculations for interpretation. One explanation of this improvement could be the possibility with the feedback to observe regularities in the decision-making of the opponent in some specific situation such as the systematic choice to play cross-court or down the line. Another explanation would be the possibility to memorize information from the opponent in the preparation of the action in which can have a predictive value for the shot direction (Wulf and Weigelt, 1997). This could be the placement of the feet or the orientation of the shoulders which are known to be a source of information for anticipation (Williams et al., 2002).

It could be interesting to replicate this protocol with less skilled players to analyse if this short training time is enough to generate an increase in the ability to anticipate such as Smeeton et al. (2005) in their field experiment. Triolet et al. (2022) demonstrated that tennis-specific tactical information could only be utilized by expert players. However, Murphy et al. (2016) showed that less expert players could also make use of the relative positioning of players on the court. Therefore, it is conceivable that less expert players in the various opponents Group might improve their anticipation skills through the use of this protocol.

It also seems important to ask if the improvement in response accuracy could be transferred to field situations. Indeed, Broadbent et al. (2015) suggested that training protocols should focus on assessing the efficacity of transfer from training to sport field. However, we can reasonably imagine that we could have some transfers as shown by Farrow and Abernethy (2002) or Williams et al. (2003). Another issue would be the question of retention. Indeed, Farrow and Abernethy (2002) showed that the post-test improvement (it was a decision time decrease) disappeared after 32 days, other more recent studies shown retention of learning (Abernethy et al., 2012). Even if the retention remains an important issue, our results suggest that such a protocol could be useful just before playing against a specific opponent. Indeed, we can identify a practical application for our protocol. It is well-known that victory in a high-level tennis match can sometimes hinge on winning just one or two more points than the opponent. Since our protocol appears to improve anticipation of the opponent's gameplay, it would be valuable to create a database of high-level players. A professional player could then engage with the protocol shortly before starting their match, potentially enabling them to perform more efficiently through better reading of their opponent's game from the very first points. The advantage of this protocol is that it does not require the coach to conduct prior analysis of the opponent's game. Furthermore, Masters (1992) demonstrated that implicit learning is more robust under stress conditions.

It is to be noted that our study also presents a number of limitations. Firstly, a tennis match unfolds as a dynamic interplay where players adapt to their opponent's responses and modify their game patterns, as well as their tactical and strategic choices. These evolutions cannot be accounted for in a laboratory study, which is not necessarily representative of what actually happens on the court, as recommended by Avilés et al. (2019) Another limitation relates to the differences in skill levels within our group of experts. Although all our players had extensive experience in tennis, not all of them were at a level to compete against a top 10 ATP player, who displayed significantly superior abilities. It would have been interesting to pair each player with an opponent of similar skill level. However, this approach would not have allowed us to compare the results across players and generalize the findings. Lastly, we hypothesized a practical application for our protocol. However, in order to use this training tool, videos of future opponents are required, which is unfortunately only feasible for high-level players.

To conclude, through our implicit training protocol, we showed that there is a possibility to use knowledge on anticipation to propose specific protocol with an applied perspective of improving the ability of experts to predict the action of a specific opponent. This opens some possibilities for further research to identify what information is used for this specific anticipation and also some future methods to train cognitive abilities in expert players.

## Data Availability

The raw data supporting the conclusions of this article will be made available by the authors, without undue reservation.
